# A progressive and refractory case of breast cancer with Cowden syndrome

**DOI:** 10.1186/s12957-022-02745-5

**Published:** 2022-09-03

**Authors:** Aiko Sueta, Masako Takeno, Lisa Goto-Yamaguchi, Mai Tomiguchi, Toko Inao, Mutsuko Yamamoto-Ibusuki, Yutaka Yamamoto

**Affiliations:** Department of Breast and Endocrine Surgery, Kumamoto University Graduate School of Medical Science, 1-1-1, Honjo, Chuo-ku, Kumamoto, 860-8556 Japan

**Keywords:** Multiple hamartoma syndrome, *PTEN*-related cancers, Hereditary disease

## Abstract

**Background:**

Cowden syndrome is a rare autosomal-dominant disease with a high risk of malignant tumors of the breast, commonly caused by germline mutations in the *PTEN* gene. Most breast cancers related to Cowden syndrome showed typically a slow-growing and favorable clinical course. Here, we report a progressive case of triple-negative breast cancer in a patient who was diagnosed with Cowden syndrome.

**Case presentation:**

A 35-year-old female with breast cancer was referred to our hospital. Histopathological examination of the tumor showed that it was triple-negative breast cancer with high proliferation marker. Preoperative positron emission tomography-computed tomography showed abnormal uptake in the left cerebellar hemisphere in addition to the right breast and axillary lymph node. Brain T2-weighted magnetic resonance imaging revealed hyperintense bands in the left cerebellar hemisphere lesion, which demonstrated a “tiger-stripe” appearance. The patient’s mother had died of endometrial cancer. Subsequently, she underwent genetic testing, leading to a diagnosis of Cowden syndrome with a pathogenic variant c.823_840del.18 at exon 8 in *PTEN*. She was treated with neoadjuvant chemotherapy of eribulin and cyclophosphamide followed by adriamycin and cyclophosphamide. However, her tumors increased after these treatments. She was immediately surgically treated and received adjuvant chemotherapy of capecitabine. Unfortunately, the cancer recurred in the lung nine months after surgery. We then administered paclitaxel and bevacizumab therapy, but the disease rapidly progressed. Consequently, the patient died due to breast cancer about three months after recurrence.

**Conclusion:**

We report an aggressive case of cancer with Cowden syndrome which was resistant to standard chemotherapy. Alteration of the phosphatidylinositol-3 kinase/Akt/mammalian target of rapamycin pathway due to inactivating PTEN protein may be associated with chemoresistance and serves as a candidate for therapeutic intervention in *PTEN*-related cancers.

## Background

Hereditary breast cancers account for 5–10% of all breast cancers, and the most common causes of the disease are mutations in the *BRCA1/BRCA2* genes [1]. In addition, several pathogenic alterations such as *TP53*, *PTEN*, *CDH1*, *PALB2*, *ATM*, and *CHEK2* are correlated with an increased risk of breast and other cancers [2–7]. Recently, the identification of hereditary disease has become increasingly important for patient management and risk-based surveillance.

Cowden syndrome is a rare autosomal dominant syndrome which is mostly caused by germline mutations in the phosphatase and tension homolog (*PTEN*) tumor suppressor gene, located on chromosome 10q23 [3]. So far, hundreds of mutations in *PTEN* have been identified [8, 9], and inactivating PTEN protein has been shown to lead to uninhibited phosphorylation of Akt, resulting in dysregulation of the mTOR pathway that is responsible for tumor survival, proliferation, and apoptosis [10, 11].

Cowden syndrome is characterized by multiple hamartomas and an increased risk of breast, thyroid, melanoma, and endometrial cancers [12], (https://raredisease.org/rare-disease/pten-hamartoma-tumor-syndrome/). There appears to be a variety of breast diseases related to Cowden syndrome, including benign disease [13], cancers [14], and sarcomas [15]. Given that the lifetime risk of developing breast cancer is around 85%, the National Comprehensive Cancer Network (NCCN) includes breast cancer as a major diagnostic criterion for Cowden syndrome, along with Lhermitte-Duclos disease, thyroid carcinoma, and macrocephaly (http://www.nccn.org/professionals/physician_gls/pdf/genetics_bop.pdf.).

According to a recent review of the literature, most breast cancers related to Cowden syndrome involve estrogen receptor (ER)-positive tumors, typically with a slow-growing and favorable clinical course [14]. On the other hand, it is reported that there are no distinguishing differences in histopathological features and clinical courses between breast cancers in patients with Cowden disease and those in the general population [16].

Here, we describe a progressive case of a 35-year-old female with triple-negative (TN) breast cancer related to Cowden syndrome, who harbored pathogenic variants in the *PTEN* gene and had little response to standard chemotherapy.

## Case presentation

**Fig. 1:**
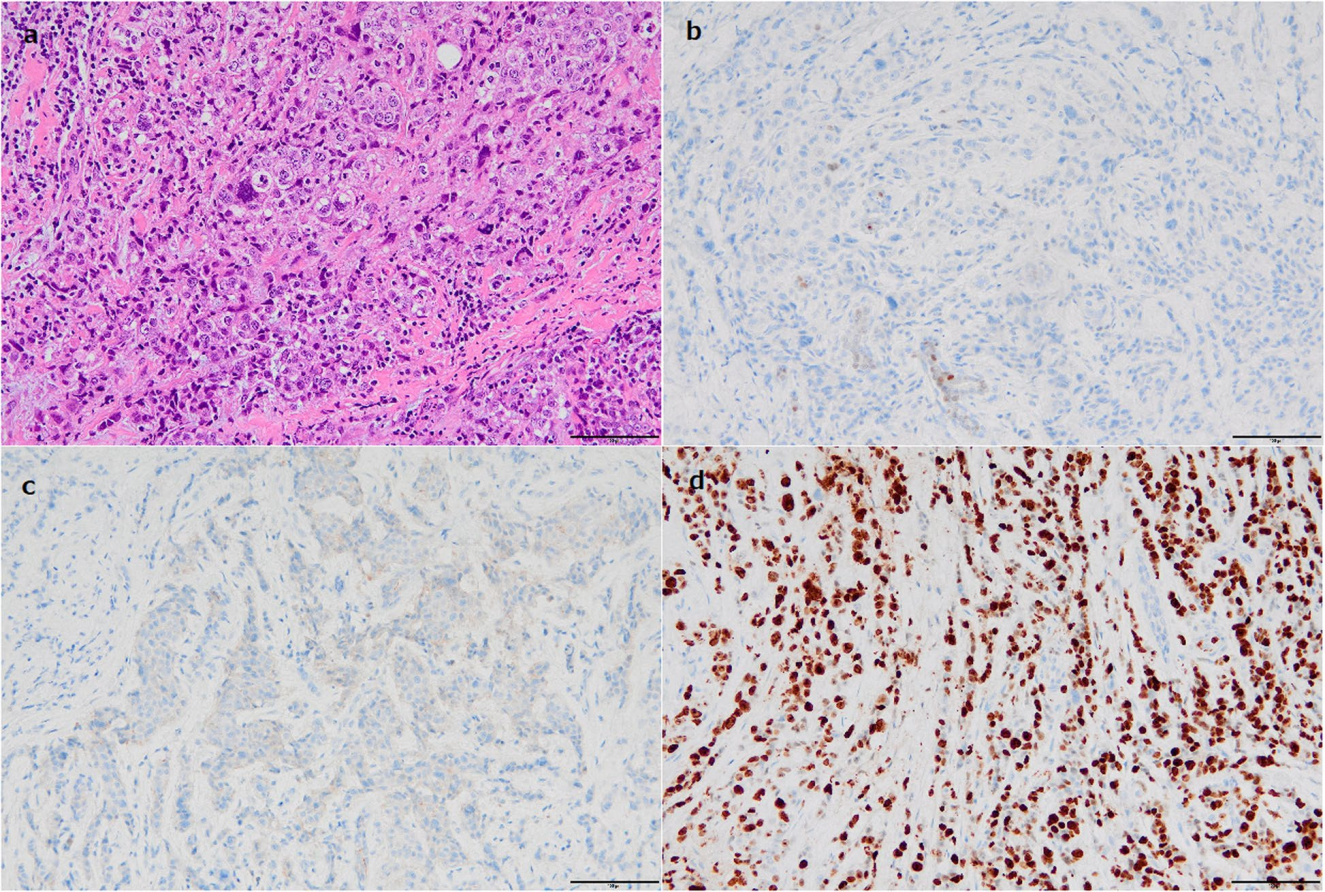
Pathological examinations of the core-biopsy specimen from the right breast before treatment (× 200 magnification). H&E staining shows invasive ductal carcinoma with high histological grade (**a**). ER staining was negative (**b**). HER2 staining was negative (**c**). Ki67 labeling index was more than 90% (**d**). Black bar represents 100 μm length

**Fig. 2:**
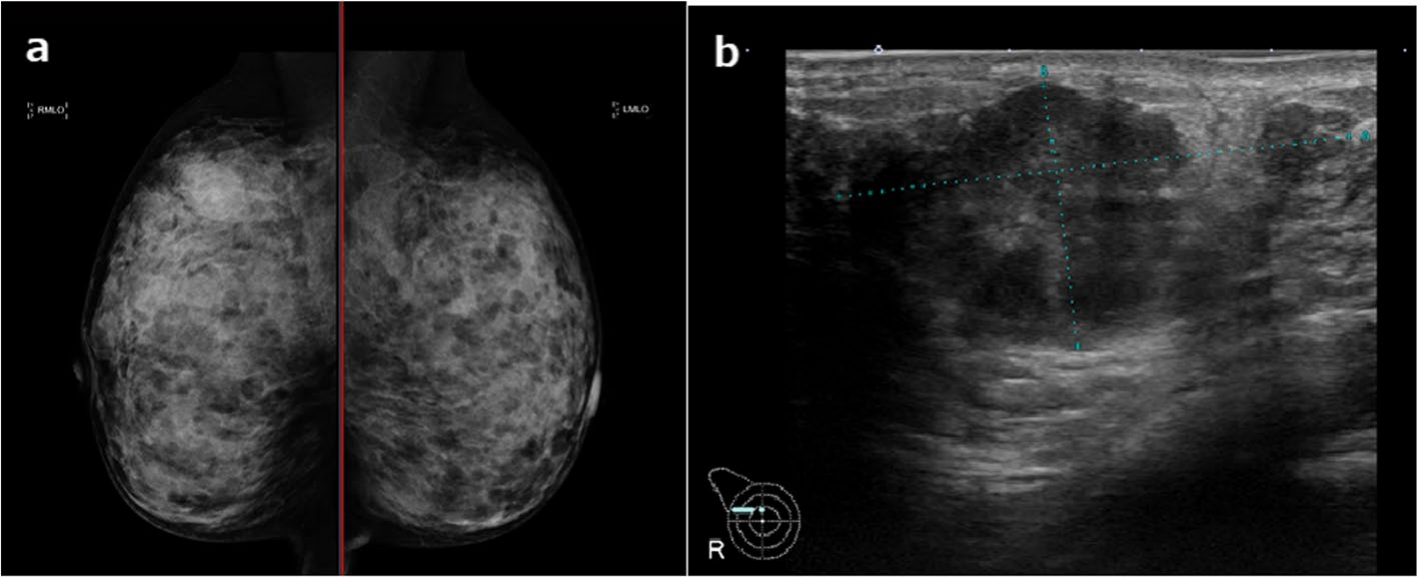
Mammography at diagnosis shows an indistinct high-density mass in the right breast (**a**). Ultrasonography identifies an irregular-shape low echoic mass in the right breast (**b**)

**Fig. 3:**
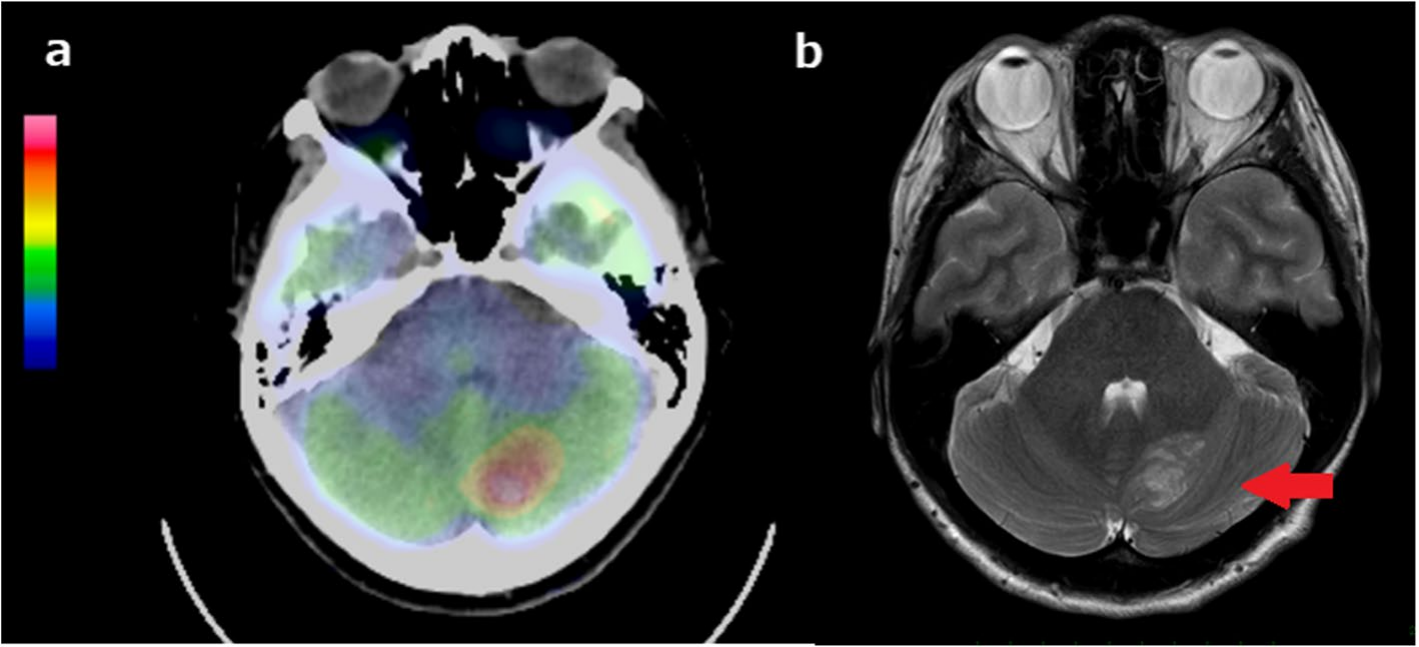
PET-CT demonstrates abnormal uptake with SUV max = 17.8 in the left cerebellar hemisphere (**a**). T2-weighted MRI reveals hyperintense bands, which suggested “tiger-stripe” appearance (red arrow) (**b**)

A 35-year-old woman was referred to our hospital with a cancer of the right breast. She had discovered a breast mass two months previously and had already been diagnosed with invasive ductal breast cancer at another hospital. A histopathological examination of the core-needle biopsy showed that it was negative for ER, progesterone receptor (PR), and human epidermal growth factor receptor (HER2). Ki-67 labeling index was more than 90% (Fig. 1). The histological grade was three. Mammography showed an indistinct high-density mass in the right breast and ultrasonography showed an irregular-shaped low echoic mass with a size of 39 × 20 × 18 mm in the upper-outer region of the right breast (Fig. 2). Screening by positron emission tomography-computed tomography (PET-CT) showed abnormal uptake in the right breast and axillary lymph node. There was no apparent distant metastasis but abnormal uptake in the left cerebellar hemisphere (Fig. 3a). Brain T2-weighted magnetic resonance imaging (MRI) revealed hyperintense bands, which demonstrated a “tiger-stripe” appearance, in the corresponding lesion (Fig. 3b). After consulting the department of neurosurgery, this finding was diagnosed as Lhermitte-Duclos disease. The patient also had asthma and schizophrenia. Her mother had died of endometrial cancer at 57 years old. There was no other family history of cancers. We suspected Cowden disease due to the findings of brain MRI. She was subsequently referred for genetic counseling and underwent genetic testing, which revealed germline alterations in the *PTEN* gene; c.823_840del.18 at exon 8 (p. 275_280del.).

**Fig. 4:**
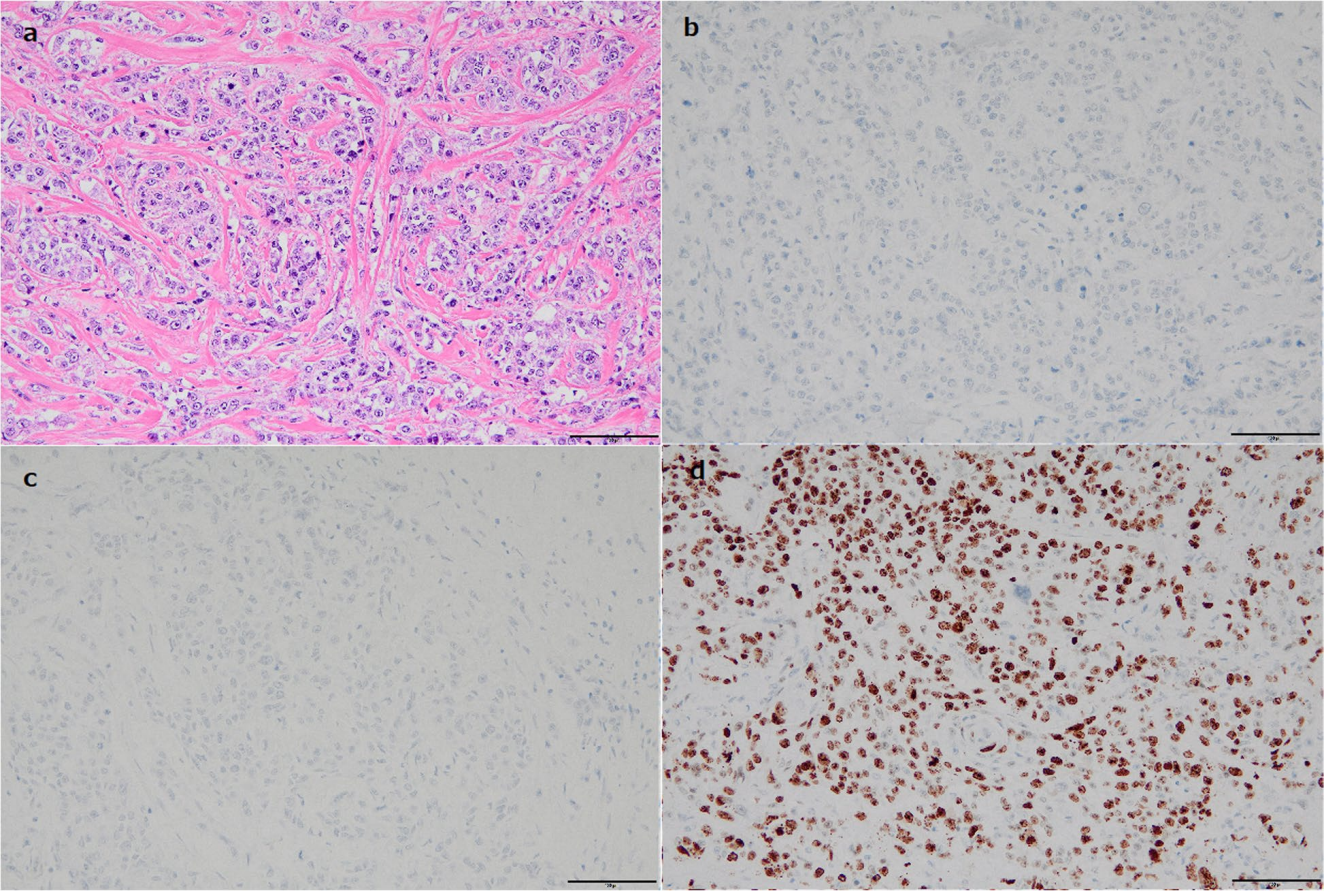
Postoperative pathological examinations of the right breast after chemotherapy (× 200 magnification). H&E staining shows remains of invasive ductal carcinoma (**a**). ER staining was negative (**b**). HER2 staining was negative (**c**). Ki67 labeling index was 90% (**d**). Black bar represents 100 μm length. Therapeutic effect of chemotherapy was grade 1a

Based on the above examination, the patient was diagnosed with TN breast cancer that was defined as the absence of ER, PR, and HER expression, at stage IIB, cT2N1M0 and received neoadjuvant chemotherapy. We administered four courses of eribulin (1.4 mg/m^2^) and cyclophosphamide (600 mg/m^2^), which was a doctor-initiated clinical trial, and subsequently one course of adriamycin (60 mg/m^2^) and cyclophosphamide (600 mg/m^2^). After these treatments, the tumor had increased in size to 50 mm and was considered clinically progressive disease. We then switched to surgery and she underwent mastectomy of the right breast and axillary lymph node dissection. Postoperative pathological examination revealed invasive ductal carcinoma, histological grade 3, and pT3 (51 mm) N3a (10/29) M0 stage IIIC. ER, PR, and HER2 were negative and Ki-67 labeling index was 90% (Fig. 4). The chemotherapeutic effect was grade 1a. After surgery, she received seven courses of adjuvant treatment with capecitabine (1800 mg × 2/day), which was based on the CREATE-X trial [17]. After seven courses of the treatment, she suffered from dyspnea, resulting in multiple lung metastases and cancerous lymphangiopathy. She had a diagnosis of recurrence of the breast cancer 9 months after surgery.

**Fig. 5:**
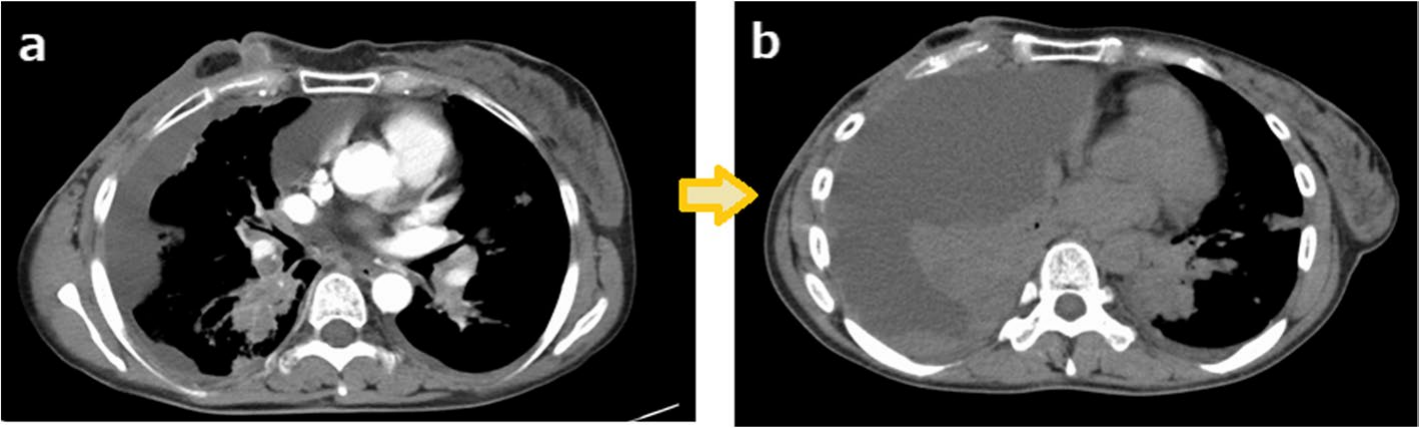
Contrast-enhanced CT performed at the time of recurrence demonstrates pleural effusion and dissemination (**a**). After one course of paclitaxel and bevacizumab treatment, pleural effusion was exacerbated and tumors in the lung were increased (**b**)

As the first-line treatment after recurrence, we administered chemotherapy of bevacizumab (10 mg/kg) and paclitaxel (90 mg/m^2^). However, after one course of treatment, pleural effusion was exacerbated and tumors in the lung increased (Fig. 5). We then tried treatment with gemcitabine (1250 mg/m^2^) as the second line of therapy, but tumors progressed rapidly, and no therapeutic effect was achieved. The patient died due to breast cancer about 3 months after recurrence.

## Discussion and conclusions

We report a progressive case of TN breast cancer with Cowden syndrome, which did not respond to standard chemotherapy and followed a rapid course to the end. Cowden syndrome is rare and the estimated incidence is 1/200,000, although it is likely to be underestimated due to difficulties in diagnosing the disease [18]. There is a need for better understanding of this rare disease, since the widespread use of cancer genomic testing has led to an increase in the chances of unexpected diagnosis.

The diagnostic criteria for Cowden disease were first described in 1995 [18] and have been modified and updated over the years to their current form. The current diagnostic criteria proposed by the NCCN include breast cancer, endometrial cancer, thyroid cancer, gastrointestinal hamartomas, Lhermitte-Duclos disease (adult), macrocephaly, macular pigmentation of the glans penis, and multiple mucocutaneous lesions, as major criteria [17, 19] (Table 1). Minor criteria include ten items such as colon cancer and renal cell carcinoma. About 80% of the patients who meet these diagnostic criteria appear to have a germline *PTEN* mutation [9]. An individual without family history of Cowden syndrome is clinically diagnosed with PTEN hamartoma syndrome if any of the following are met: three or more major criteria, but one must include macrocephaly, Lhermitte-Duclos disease, or gastrointestinal hamartomas, or two major and three minor criteria [19]

**Table 1:** Revised PTEN hamartoma tumor syndrome clinical diagnostic criteria

Major Criteria	Minor Criteria
Breast Cancer	Autism spectrum disorder
Endometrial cancer (epithelial)	Colon cancer
Thyroid cancer (follicular)	Esophageal glycogenic acanthoses (>3)
GI hamartomas	Lipomas (>3)
-including ganglioneuromas, but excluding hyperplastic polyps; >3	Intellectual disability (ie. IQ <75)
Lhermitte-Duclos disease (adult)	Renal cell carcinoma
Macrocephaly	Testicular lipomatosis
->97th percentile: 58cm for females, 60 cm for males	Thyroid cancer (papillary or follicular variant of papillary)
Macular pigmentation of the glans penis	Thyroid structural lesions (eg, adenoma, multinodular goiter)
Multiple mucocutaneous lesions (any of the following):	Vascular anomalies (including multiple intracranial developmental venous anomalies)
-Multiple trichilemmomas (>3, at least one biopsy proven)	
-Acral keratoses (>3 palmoplantar keratic pits and/or acral hyperkeratotic papules)	
-Mucocutaneous neuromas (>3)	
-Oral papillomas (particularly on tongue and gingiva), multiple (>3) OR biopsy proven OR	

In the present case, the patient did not strictly meet the clinical diagnostic criteria. We had suspected Cowden syndrome because of the findings of Lhermitte-Duclos disease, although the patient had no strong family history of cancers and no manifestations related to *PTEN* mutations, such as macrocephaly or mucocutaneous lesions. A family history of cancers is often unclear due to the high frequency of de novo PTEN germline mutation [20]. We conducted risk assessment and counseling of genetic testing, and finally, the patient decided to undergo the genetic test. If breast cancer patients have macrocephaly, which is one of the useful clinical features related to *PTEN* mutations [21], clinicians need to suspect potential Cowden syndrome.

Lhermitte-Duclos disease is a rare and slow-growing hamartoma, which appears to be strongly related to *PTEN* mutations and included as a major criterion for the diagnosis of Cowden’s syndrome [19, 22]. When the patient has some findings of Lhermitte-Duclos disease, the prevalence of affected *PTEN* is up to 80% [19]. There were no other intracranial findings in the present case, but Dhamija et al. have emphasized that it is important to recognize that other neuroimaging abnormalities such as meningiomas, venous anomaly, and cavernous malformations may warrant suspicion of Cowden disease in the clinical setting [23].

By genetic testing, we found deletion of the *PTEN* gene at a length of 18 bases at exon 8 in the present case. More than 80% of patients with Cowden syndrome have been found to have a germline *PTEN* pathogenic variant [12], although there were no correlations between mutation types and tumor types [24]. These variants include missense, nonsense, and splice site variants, small deletions, insertions, and large deletions [12]. Most pathogenic variants in *PTEN* appear to be unique, but several recurrent variants have been reported such as c.388C>T, c697C>T, and c1003C>T [25]. The germline mutation spectrum in *PTEN* is broad, although two thirds of mutations occur in exon 5, 7, and 8 [26]. Deletions in the *PTEN* gene, such as that in the present case, are less common than single base pair alterations [25, 27], and we found only three variants of deletion in the *PTEN* gene at exon 8 according to published data (https://databases.lovd.nl/shared/genes/PTEN). Variants within the core catalytic motif in *PTEN* (at exon 5) commonly abrogate pan-phosphatase activity [28]. It is unknown to what extent some deletions at exon 8 contribute to lipid phosphatase activity leading to enhanced phosphatidylinositol-3 kinase (PI3K)/Akt/mammalian target of rapamycin (mTOR) signaling.

According to the literature, breast cancers related to Cowden syndrome tend to grow slowly and have a favorable clinical course reflecting their positive ER status [14]. However, the present case involved tumors with the TN subtype which has a poorer prognosis than other subtypes [29]. Indeed, most standard chemotherapy, including an anthracycline and taxane-containing regimen, was ineffective, but rather the tumors were found to have grown. Such treatment is very effective for a minority of TN patients, but there are many TN patients for whom it is less effective resulting in a poor prognosis [29]. In addition to the aggressive nature of TN disease, the poor prognosis may be attributable to activation of the PI3K/Akt/mTOR pathway due to the loss of function of PTEN*.* PI3K/Akt pathway is one of the critical pathways that control cell survival, migration, proliferation, and cell cycle progression, and PTEN serves as tumor-suppressor by dephosphorylating phosphatidylinositol-3,4,5-triphosphate (PIP3) to phosphatidylinositol-4,5-bisohosphatase (PIP2) [25]. The PI3K leads to phosphorylation of Akt, an effect of which is also antagonized by PTEN [30]. Alteration of the PI3K/Akt/mTOR pathway is associated with chemoresistance and serves as a candidate for therapeutic intervention in *PTEN*-related cancers and efforts to identify therapeutic strategies that focus on the PI3K pathway have been made. Sirolimus, which is an mTOR inhibitor, has been used in a phase II clinical trial for *PTEN* hamartoma tumor syndrome (NCT00971789) [31]. A therapeutic effect in decreasing mTOR signaling and evidence of improvement of symptoms were observed [31]. Besides, upstream components of the PTEN signaling pathway, such as PI3K and Akt, offer promise for treatment of *PTEN*-related cancers. In patients with advanced endometrial cancer with alterations of *PTEN*, samotolisib, which is a dual PI3K/mTOR inhibitor, demonstrated modest single-agent efficacy [32]. To date, several preclinical and clinical trials using PI3K/Akt inhibitors are underway worldwide [25, 33, 34].

In conclusion, we report a rare case of breast cancer with Cowden syndrome. TN-type breast cancer associated with Cowden syndrome may have a more advanced course than usual. Cowden syndrome is rare and still poorly understand compared to other familial cancers such as hereditary breast and ovarian cancer. We need to understand morphologic and biological features of the disease in order to achieve precise clinical management and overcome resistance to treatment.

## Data Availability

The data generated during current study are not publicly available.

## Abbreviations

ER: Estrogen receptor
HER2: Human epidermal growth factor receptor
MRI: Magnetic resonance imaging
NCCN: National Comprehensive Cancer Network
PET-CT: Positron emission tomography-computed tomography
PR: Progesterone receptor
PTEN: Phosphatase and tension homolog
TN: Triple-negative
